# Novel insights into the pervasive role of RNA structure in post-transcriptional regulation of gene expression in plants

**DOI:** 10.1042/BST20210318

**Published:** 2021-08-26

**Authors:** Huakun Zhang, Yiliang Ding

**Affiliations:** 1 Key Laboratory of Molecular Epigenetics of the Ministry of Education, Northeast Normal University, Changchun 130024, China; 2 Department of Cell and Developmental Biology, John Innes Centre, Norwich Research Park, Norwich NR4 7UH, U.K.

**Keywords:** plant RNA biology, post-transcriptional regulation, RNA structure, RNA structure profiling

## Abstract

RNA folding is an intrinsic property of RNA that serves a key role in every step of post-transcriptional regulation of gene expression, from RNA maturation to translation in plants. Recent developments of genome-wide RNA structure profiling methods have transformed research in this area enabling focus to shift from individual molecules to the study of tens of thousands of RNAs. Here, we provide a comprehensive review of recent advances in the field. We discuss these new insights of RNA structure functionality within the context of post-transcriptional regulation including mRNA maturation, translation, and RNA degradation in plants. Notably, we also provide an overview of how plants exhibit different RNA structures in response to environmental changes.

## Introduction

In the past decades, research into gene regulation has been focused on DNA and proteins. Little attention has been paid to RNA since RNA was regarded as the intermediator between DNA and proteins. However, numerous studies have shown that mRNA levels only partially correlate with protein levels [1,2]. More recently, this has led to an increasing interest in post-transcriptional gene regulation in years. RNA molecules participate in every step of post-transcriptional regulation of gene expression. RNA folding is an intrinsic property that impacts the whole post-transcriptional processes from RNA splicing, polyadenylation, and translation, through to RNA degradation [3].

Since 2010, several high-throughput RNA structure profiling methods have transformed the scope of RNA structure studies, enabling genome-wide structurome analyses [4–10]. The first genome-wide *in vitro* RNA structure profiling method, parallel analysis of RNA structure (PARS), was achieved by coupling enzymatic probing with high-throughput sequencing [7]. Enzymatic probing of RNA structure is based on the properties of different ribonucleases (RNases) cleaving either single-stranded (ss) RNA regions or double-stranded (ds) RNA regions to indicate RNA base-pairing status [7]. Next, *in vivo* RNA structure profiling methods merged chemical probing methods with high-throughput sequencing. These chemical probing methods include the DMS-based method, which uses dimethyl Sulfate to probe for single-stranded A and C nucleotides; and the SHAPE (Selective 2′-Hydroxyl Acylation analyzed by Primer Extension)-based method, which utilizes a variety of chemicals, e.g. 2-methylnicotinic acid (NAI), to probe the single-strandedness of all four RNA nucleotides [10]. The emergence of these advanced technologies has significantly progressed the field of RNA structure research, facilitating many new insights into RNA structure functionalities.

In this review, we summarize how new technologies that reveal the RNA structurome have provided unique insights into the functional roles of RNA structure regarding the whole post-transcriptional process — from mRNA maturation, translation, through to RNA degradation. We also include additional findings relating to ancillary roles of RNA structure in other post-transcriptional regulatory pathways. In parallel, we provide an overview of several exciting studies that focus on how plants adopt RNA structures to facilitate regulatory responses to various environmental stresses including heat, light and salt stress.

## The role of RNA structure in mRNA maturation

Following transcription, nascent RNAs undergo a maturation process that involves intron splicing, 5′ capping and 3′ polyadenylation to create mature mRNA that can be exported to the cytoplasm [11,12]. 5′ capping in eukaryotes is the process that links an N^7^-methylated guanosine to the first nucleotide of the RNA via a reverse 5′ to 5′ triphosphate linkage [13]. RNA splicing is the process that the spliceosome removes introns from messenger RNA precursors (pre-mRNAs) [12]. The spliceosome is a complex molecular machine involving five small nuclear RNAs (snRNAs) and ∼100 proteins [12]. The U1 and U2 small nuclear ribonucleoproteins (snRNPs) recognize the 5′ splice site (5′ss) and the branch point (BP) sequence, respectively, to form the prespliceosome, which then associates with the U4/U6.U5 tri-snRNP to form the full spliceosome for removing the intron [12]. 3′ polyadenylation is mediated by a large complex of proteins, cleavage and polyadenylation specificity factors (CPSFs), where CPSFs recognizes the polyadenylation signal in the pre-mRNA, processes the RNA substrate, and adds as many as ∼200 adenosines [14]. Thus, mRNA maturation is the first key stage in controlling post-transcriptional RNAs and considerable effort has been made to identify the large number of proteins involved this multi-step process [11,12]. It is unclear how mRNA processing sites, such as polyadenylation and splice sites, are recognized and distinguished from surrounding regions with similar sequence content [15,16]. To address this issue, a previous study utilized RNase enzymes to generate the *in vitro* RNA structurome in *Arabidopsis thaliana* nuclear RNAs [17]. They found that the 5′ end of introns were more double-stranded compared with upstream exons while the 3′end of introns were more single-stranded compared with upstream intron regions [17]. No significant structure signatures were identified for polyadenylation sites [17].

**Figure 1.**
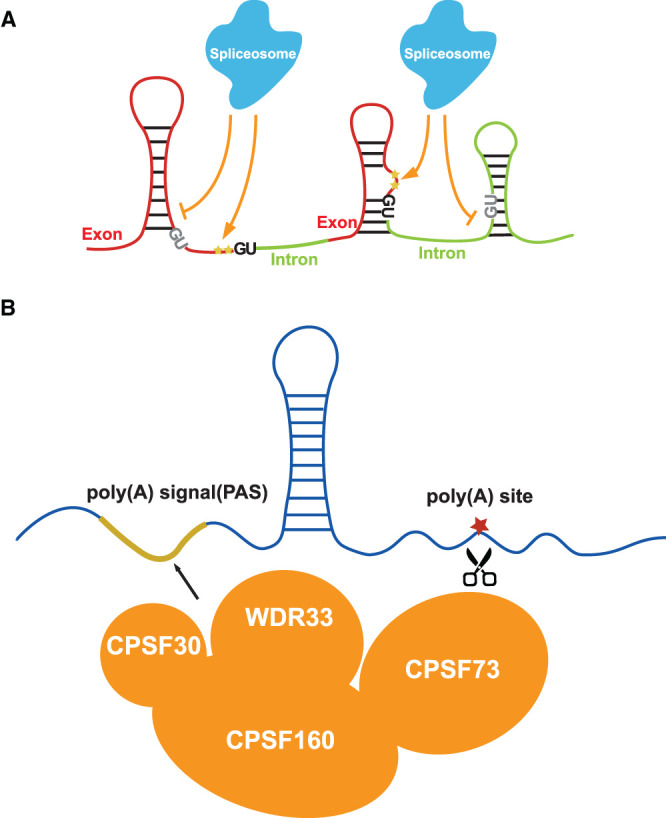
New insights of RNA structure functionality in mRNA maturation. (**A**) A two-nucleotide single-stranded RNA structure feature (yellow stars) upstream of 5′splice sites (5′ss, upstream of the first two-dinucleotide ‘GU' in the intron) is strongly associated with splicing. If two nucleotides upstream of GU (in dark) are single-stranded, the splicing is switched on. If two nucleotides upstream of GU (in grey) are double-stranded, the splicing is switched off. (**B**) Two close-by single-stranded regions across polyadenylation sites are strongly associated with both polyadenylation and alternative polyadenylation events. The first single-stranded region (highlighted in the yellow line) is from −28 nt to −17 nt upstream of the polyadenylation (poly(A)) sites, overlapped with the PAS motif. This region might serve as the recognition site for RNA binding proteins such as CPSF30, CPSF160 and WDR33. The other region (red star) is from −4 nt to +1 nt across the poly(A) site which might facilitate the cleavage of CPSF73.

In contrast, a recent *in vivo* RNA structure study using *in vivo* SHAPE chemical probing on *Arabidopsis thaliana* nuclear RNAs, revealed various structural features [18], indicating that earlier *in vitro* RNA structure approaches were not able to reflect the proper folding status of RNAs in living cells. The *in vivo* nuclear RNA structurome revealed a two-nucleotide single-stranded RNA structure feature upstream of 5′ss that is strongly associated with splicing and the selection of alternative 5′ss, while the single-strandedness of branch sites is also associated with 3′splice site (3′ss) recognition [18] (Figure 1A). Interestingly, experimental tuning of the two-nucleotide single-stranded RNA structure feature upstream of 5′ss was shown to be sufficient to change splicing fate [18], suggesting that fine RNA structure features may have evolved to facilitate splicing recognition. Notably, U1 snRNA base-pairs have a total of nine nucleotides (from −3 to +6 region of 5′ss) across 5′ss [19,20]. Once the 5′ss is recognized by base-pairing with U1 snRNA, the spliceosome is assembled onto the intron region and the 5′ss-U1 interaction is replaced by interactions of 5′ss with U5 snRNA (from −3 to −1 region of 5′ss). Thus, this two-nucleotide single-stranded RNA structure feature of the −1 and −2 positions upstream of 5′ss may be involved in either the interaction with U1 snRNA or the later interaction with U5 snRNA. Further biophysical studies are required to provide mechanistic insight of the interaction between this RNA structure feature and snRNAs.

For polyadenylation, a structure feature comprising of two close-by single-stranded regions across polyadenylation sites were identified to be strongly associated with both polyadenylation and alternative polyadenylation events [18] (Figure 1B). The first single-stranded region was from −28 nt to −17 nt upstream of the polyadenylation (poly(A)) sites and the other region was from −4 nt to +1 nt across the poly(A) sites [18]. Interestingly, the first single-stranded region overlapped with the conventional polyadenylation signal (PAS) motif ‘AAUAAA' [18]. In plants, ∼10% of *Arabidopsis* genes contain the PAS sequence motif [21], suggesting that single-stranded RNA structure features might be adopted as an additional signature for unconventional polyadenylation recognition beyond sequence. The maintenance of single-strandedness across PAS sites might allow the recognition by single-stranded RNA binding proteins such as CPSF30, CPSF160 and WDR33, which are crucial during polyadenylation [22]. The other single-stranded region might facilitate endonucleolytic cleavage at the poly(A) sites catalyzed by RNA binding proteins, such as CPSF73 [23]. Further RNA structure studies could be conducted in mutants of these RNA binding proteins to assess the structural requirements for interactions with individual proteins, e.g. the length of single-strandedness.

## The role of RNA structure in translation

Following nascent mRNA processing, mature mRNAs are exported to the cytoplasm where they undergo translation. Both *in vitro* and *in vivo* RNA structure profiling in *Arabidopsis* showed that a single-stranded region upstream of the start codon was strongly associated with a high translation efficiency, suggesting that this feature might facilitate ribosome binding and translation initiation [5,24]. Furthermore, a triplet periodic trend of DMS reactivities was observed in the CDS, but not UTRs, for mRNAs with high translation efficiency, however this feature was absent for mRNAs with low translation efficiencies [5]. This triplet periodic pattern of RNA structure features might be associated with the triplet movement of the ribosome during translation [25]. Notably, similar patterns were also observed for rice (*Oryza sativa*) [26]. These global RNA structure patterns associated with translation efficiency suggested that RNA structure may have a pervasive role in translational regulation [5,24,26].

**Figure 2.**
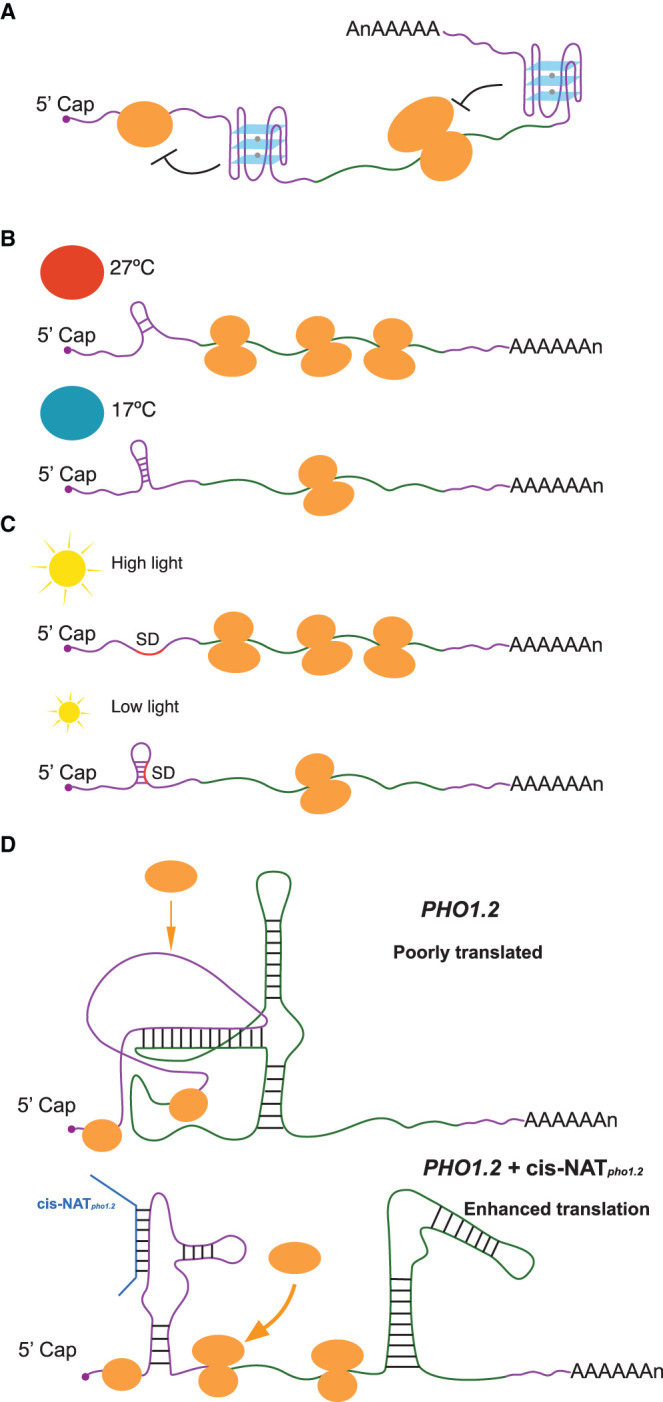
New insights of RNA structure functionality in translation. (**A**) RNA G-quadruplexes localized in either 5′UTR or 3′UTR are capable of repressing translation. (**B**) An RNA hairpin within the 5′ UTR of *PIF7* was changed into an alternative conformation at higher temperature, leading to increased translation of *PIF7*. (**C**) The translation initiation region of *psbA* was changed to more single-strandedness under high light stress, increasing the translation of *psbA*. (**D**) A localized sense–antisense inter-molecular interaction between *PHO1.2* and *cis-NATpho1.2* rearranged the RNA structure to allow more access of 60S to the translation initiation site, enhancing *PHO1.2* translation. Small orange circles represent small ribosomal subunits, whilst big orange circles indicate large ribosomal subunits.

In addition to RNA secondary structures, RNAs also fold into complex tertiary structures [27,28]. One of the well-known tertiary structures is RNA G-quadruplex (RG4), which is folded with guanine-rich (G-rich) sequences and consists of two or more layers of G-quartets involving both Hoogsteen and Watson–Crick base pairs [27,28]. An *in vitro* RNA structure study reported the first highly conserved plant RG4 located in the 5′ untranslated region (UTR) of *ATAXIA TELANGIECTASIA-MUTATED AND RAD3-RELATED* (*ATR*) and revealed that translation is inhibited when stable GQS structures are formed [29] (Figure 2A). Another study identified an RG4-mediated translational regulatory module for phloem development, whereby the zinc-finger protein JULGI(JUL) binds to the RG4 site in the 5′UTR of central regulators of phloem formation *- SUPPRESSOR OF MAX2 1-LIKE4/5* (*SMXL4/5*) [30,31]. This direct binding between JUL and the RG4 site was shown to repress the translations of *SMXL4/5*, thus restricting phloem differentiation [30,31] (Figure 2A). Interestingly, this RG4 is exclusively conserved in vascular plants, suggesting this RG4 may have evolved alongside the emergence of phloem during land plant evolution [30,31].

A more recent genome-wide study established a novel RG4 profiling method: selective 2′-hydroxyl acylation with lithium ion-based primer extension coupled with high throughput sequencing, SHALiPE-Seq. SHALiPE-Seq is based on the preferential modification of the last G in G tracts of folded RG4s by NAI [32]. SHALiPE-Seq was used to determine hundreds of RNA G-quadruplex structures are strongly folded in both *Arabidopsis* and rice [32]. This approach provided the first direct evidence of RNA G-quadruplex formation in living eukaryotic cells. In addition to genome-wide observations, an individual RG4 identified in the 3′UTR of gene *HIRD11*, which encodes a KS-type dehydrin, was shown to be capable of suppressing its own translation to modulate plant growth and development [32] (Figure 2A). In contrast, *in vivo* experiments carried out for both yeast and mice have not detected RG4 structures [33]. It is tempting to speculate that, as sessile organisms, plants may therefore have evolved RG4 structures as an extra layer in regulating translation in response to evolution.

The sessile nature of plants makes them more susceptible than animals to varying environmental conditions, such as changes in temperature and light [34]. A recent study in *Arabidopsis* showed that the transcript encoding the bHLH transcription factor PIF7 undergoes a direct increase in translation in response to warmer temperature [35]. An RNA hairpin within its 5′ UTR was determined and changed into an alternative conformation at a higher temperature, leading to increased translation of *PIF7* [35] (Figure 2B). Similar hairpin sequences were identified in other RNAs, including *WRKY22* and the key heat shock regulator *HSFA2*, suggesting that this hairpin structure-mediated translational regulation may be a conserved mechanism enabling plants to respond and adapt rapidly to high temperatures [35]. Another recent study exposed *Arabidopsis* to high light stress which induced translation of *psbA* mRNA encoding the D1 subunit of photosystem II [36]. *In vivo* RNA structure analysis revealed this increase in translation was due to a change to more single-strandedness across the translation initiation region of *psbA* [36] (Figure 2C). Other plastid genes with weak Shine-Dalgarno sequences (SD) exhibited similar RNA structure-mediated translational regulation, suggesting this maybe a general regulatory mechanism for translation regulation of plastid genes in plants [36].

An antisense-mediated regulatory mechanism was identified in rice, where translation of the phosphate transporter *PHOSPHATE1.2* (*PHO1.2*) translation was enhanced under the phosphate-deficient conditions when the expression of its antisense RNA (*cis-NATpho1.2*) increased [37]. A recent RNA structure analysis revealed that a high GC region in *PHO1.2* downstream of its start codon generated a strong structure which inhibited binding of the 60S subunit to the 40S [38]. In the presence of *cis-NATpho1.2*, a localized sense–antisense inter-molecular interaction rearranged this inhibitory structure to allow the 60S access to the translation initiation site, increasing 80S initiation complex formation and subsequently enhancing *PHO1.2* translation [38] (Figure 2D).

## The role of RNA structure in RNA degradation

RNA structures can comprise single-stranded regions, hairpin loops, internal loops, bulges and so forth. These structural motifs have different thermodynamic properties that together determine the overall RNA stability [10,39]. A recent genome-wide RNA structure study using DMS-based RNA structure profiling in rice revealed that RNA structures were globally unfolded after 10 min of heat shock at 42°C from 22°C [40]. Unexpectedly, this global RNA structural alteration was not associated with translational changes, but instead with RNA abundance, suggesting that mRNA unfolding in response to heat stress may facilitate access to RNA degradation machinery [40]. A similar study in *Arabidopsis* found that RNA structures in both shoot and root were globally refolded in response to salt stress, leading to an inverse change of RNA abundance [41]. In contrast, protein interaction profile sequencing (PIP-seq), a method that simultaneously identifies protein-bound regions on a transcriptome-wide scale to examine global patterns of *in vitro* RNA secondary structure, reached the opposite conclusion following systemic salt stress in *Arabidopsis* [42]. They observed that N^6^-methyladenosine (m^6^A) RNA changes anti-correlated with alterations of RNA secondary structures in response to salt stress [42]. Interestingly, the salt-specific m6A deposition and the associated weak RNA secondary structure resulted in increases in mRNA stability [42]. These contrasting results might be due to the different time periods employed for salt treatment [41,42]. The first study included a 48 h exposure to 100 mM NaCl stress, while the systematic salt stress treatment in the latter study involved slowly increasing the NaCl concentration in the watering solution in 50 mM increments every three days, starting from 50 mM NaCl and rising to a final concentration of 150 mM NaCl, followed by a 10-day treatment of 150 mM NaCl [41,42].

**Figure 3.**
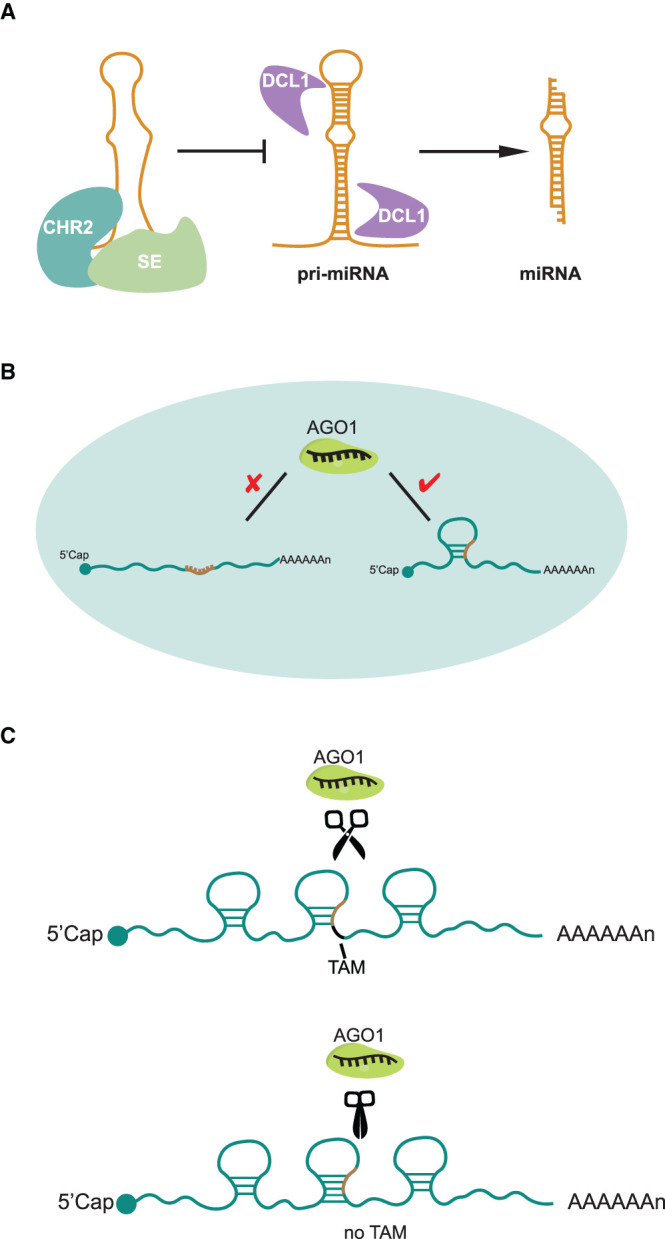
New insights of RNA structure functionality in miRNA-mediated RNA degradation. (**A**) CHR2 interacted with SE accessed pri-miRNAs and remodelled their RNA secondary structures, preventing the formation of hairpin structures and subsequently inhibiting the pri-miRNA processing by DCL1 that is required to generate mature miRNAs. (**B**) miRNA target sites were not structurally accessible for miRISC binding prior to cleavage in living cells. (**C**) The single-strandedness of the two nucleotides immediately downstream of the target site, TAM, is capable of triggering miRNA cleavage.

The microRNA (miRNA)-mediated gene silencing pathway including both translation inhibition and RNA degradation is highly specific in plant but poorly specific in animals [43]. MiRNAs are ∼21 nucleotide RNAs derived from primary precursors of miRNAs (pri-miRNAs) which contain imperfect foldback hairpin structures [43]. miRNAs are then loaded onto ARGONAUTE proteins (AGO) to form functional post-transcriptional gene silencing effector complexes, termed miRISCs (miRNA-Induced Silencing Complexes) [43]. For the miRNA-mediated RNA degradation, each miRISC is guided by the miRNA to bind targeted RNAs through sequence complementarity and to trigger cleavage [43]. A recent DMS-based RNA structure study revealed that CHR2, the ATPase subunit of the large switch/sucrose non-fermentable (SWI/SNF) complex, accessed pri-miRNAs through interaction with the microprocessor component Serrate (SE). This interaction remodelled their RNA secondary structures, preventing the formation of hairpin structures and subsequently inhibiting the pri-miRNA processing by DCL1 (Microprocessor–Dicing complex includes Dicer-like 1) that is required to generate mature miRNAs [44] (Figure 3A). A new SHAPE-based RNA structure profiling method, CAP-STRUCTURE-seq, was developed to capture *in vivo* structures of mRNAs before cleavage using the terminator exonuclease treatment for the enrichment of intact RNAs [45]. This method was recently used to reveal a regulatory mechanism of targeted mRNA structure during miRNA-mediated RNA degradation in *Arabidopsis thaliana* [45]. Surprisingly, this approached revealed that miRNA target sites were not structurally accessible for miRISC binding prior to cleavage *in vivo* [45] (Figure 3B). Instead, unfolding of the target site structure is critical to the miRISC activity *in vivo* [45]. Furthermore, the single-strandedness of the two nucleotides immediately downstream of the target site, named Target Adjacent nucleotide Motif (TAM), is capable of triggering miRNA cleavage but not miRNA binding, thus uncoupling target site binding from cleavage [45] (Figure 3C). These studies demonstrate a pervasive role for RNA structure in miRNA-mediated RNA degradation.

## The role of RNA structure in other regulatory pathways

**Figure 4.**
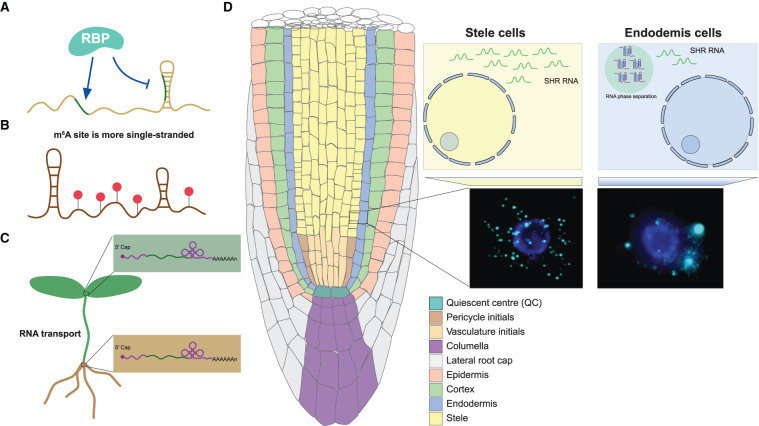
New insights of RNA structure functionality in other regulatory pathways. (**A**) RBP binding sites tend to be more single-stranded. (**B**) m^6^A association is likely to alter RNA structure into more single-strandedness. Red circles represent m^6^A modifications. (**C**) mRNAs containing a tRNA-derived structural motif can be transported intercellularly more efficiently. (**D**) *SHR* RNA contained a strongly folded RG4 structure which was shown to trigger RNA-driven phase separation in endodermis cells.

RNA binding proteins (RBPs) participate in every step of post-transcriptional regulation of gene expression and interact with their targeted RNAs in a sequence- and structure- specific manner [46]. A previous nuclear PIP-seq study found that RBP binding sites tend to be more single-stranded [17] (Figure 4A). A similar study in plant root hair and non-hair cells uncovered that distinct structural and protein binding patterns exist across the transcriptomes of these cell types. This revealed differential RNA binding protein (RBP) recognition sites, suggesting that cell-type specific RNA structures may facilitate cell-type specific interactions with RBPs [47].

Modification of RNA plays an important role in mRNA metabolism and translation [48]. m^6^A has been identified as the most abundant RNA modification in eukaryotic mRNAs [48] with recent genome-wide studies showing an enrichment of m^6^A around the start codon, stop codon and 3′UTR region in *Arabidopsis* [49,50]. This enrichment was well-correlated with single-stranded regions identified from RNA structure profiling [5,45] (Figure 4B). Similar correlations were observed in rice where higher m^6^A modification sites tend to have less RNA structure [26]. Therefore, m^6^A association is likely to alter RNA structure to favour single-strandedness and suggests fascinating functions for RNA modification in post-transcriptional gene regulation.

mRNA transport directly affects the potential of transported mRNA to be translated into proteins in target tissues [10]. A recent study combining both phenotypic and enzymatic assays on grafted plants revealed that mRNAs containing a stem-bulge-stem–loop tRNA-derived structural motif can be transported intercellularly more efficiently [51] (Figure 4C). This study demonstrated that RNA structure can also facilitate RNA mobility for intercellular communication across plants.

Phase separation is suggested as a means for ensuring appropriate molecular levels in the cells for tight regulation of gene expression [52]. A recent study discovered distinct mRNA distribution patterns of a key plant root cell identity gene SHORT ROOT (*SHR*) between different cell types using single molecule RNA FISH (smFISH) [53]. They observed dot-shaped smFISH signals of single *SHR* mRNA in stele cells, while aggregated, phase separation-like signals for *SHR* RNA were observed in neighbouring endodermis cells [53] (Figure 4D). Interestingly, *SHR* RNA contained a strongly folded RG4 structure which was shown to trigger RNA-driven phase separation *in vitro* [53]. Therefore, RNA may adopt specific RNA structure motifs, such as RG4 structures to trigger and/or maintain RNA-driven phase separation for modulating and/or affecting post-transcriptional regulations such as translation.

## Concluding remarks and future direction

New methods for studying RNA structure have resulted in tremendous progress being made in our knowledge of RNA structure functionality at every step of post-transcriptional regulation of gene expression in plants. However, limitations posed by short read sequencing platforms still pose challenges for obtaining accurate profiles of full-length structural landscapes and distinguishing structures in shared regions between isoforms. RNA structural heterogeneity is another major challenge that must be overcome in the future to improve the accuracy of RNA structure predictions. Despite efforts being made to partially resolve these issues indirectly through statistical modelling and machine learning methods [54–56], direct measurements would be greatly beneficial for distinguishing distinct RNA isoformic and conformational structures. In addition to heat, light and salt stress, plants face other environmental challenges, such as changes in acidity, drought (crowding), heavy metal stress etc., [34]. These factors have been shown to affect RNA structures [3,57], thus suggesting significant roles for RNA structures regarding environmentally induced post-transcriptional gene regulation. Improved capability for more comprehensive genome-wide RNA structure studies in multiple plant species, could enable the conservation and divergence of RNA structures to be assessed across diverse natural variants and diverse plant species. These studies should provide enhanced scope for exploring the potential for habitat and evolutionary selection at the RNA structure level. Furthermore, individual relationships between RNA structure and RNA binding proteins could be identified for phenotypic functional assessment. Similar assessments could also address individual relationships between RNA structure and RNA modification, and/or between RNA structure and RNA phase separation. This deeper understanding of the pervasive role in post-transcriptional regulation will require systematic molecular, genetic, and physiological functional measurements. Ultimately, results from such studies may have the potential to aid the development of strategies to manipulate RNA structure-mediated regulatory mechanisms to improve plant growth and environmental fitness.

## Perspectives

- Importance of the field. Functional roles of RNA structure in post-transcriptional gene regulation provide novel mechanistic insights in regulating gene expression.
- Current thinking. New insights from recent RNA structure studies have revealed a pervasive role for RNA structure in every step across post-transcriptional processes including mRNA maturation, translation, and RNA degradation. Plants adopt these functional RNA structures as key regulators in response to environmental changes.
- Future direction. Technological advances that distinguish distinct RNA isoformic and conformational structures could improve structure predictions. Comprehensive studies on RNA structure-mediated regulatory mechanisms in response to environmental challenges could also shed light on how RNA structure may be fine-tuned to modulate plant growth and development in response to varying environmental conditions. This has the potential to inform crop improvement strategies for mitigating the global impact on crops due to climate change.

## Abbreviations

3′ss: 3′ splice sites
5′ss: 5′ splice sites
m^6^A: N^6^-methyladenosine
miRISCs: miRNA-induced silencing complexes
PAS motif: polyadenylation signal motif
Poly (A): polyadenylation
pri-miRNAs: primary precursors of miRNAs
RBPs: RNA binding proteins
RG4: RNA G-quadruplex
SHALiPE-Seq: selective 2′-hydroxyl acylation with lithium ion-based primer extension coupled with high throughput sequencing
SHAPE: Selective 2′ hydroxyl acylation analyzed by primer extension
UTR: untranslated region
